# 5-{[(*E*)-2-(4-Iodo­phen­yl)hydrazinyl­idene]meth­yl}thio­phene-2-carbaldehyde

**DOI:** 10.1107/S1600536809055172

**Published:** 2010-01-09

**Authors:** Solange M. S. V. Wardell, Geraldo M. de Lima, Edward R. T. Tiekink, James L. Wardell

**Affiliations:** a CHEMSOL, 1 Harcourt Road, Aberdeen AB15 5NY, Scotland; b Departamento de Quimica, ICEx, Universidade Federal de Minas Gerais, 31270-901 Belo Horizonte, MG, Brazil; c Department of Chemistry, University of Malaya, 50603 Kuala Lumpur, Malaysia; d Centro de Desenvolvimento Tecnológico em Saúde (CDTS), Fundação Oswaldo Cruz (FIOCRUZ), Casa Amarela, Campus de Manguinhos, Av. Brasil 4365, 21040-900 Rio de Janeiro, RJ, Brazil

## Abstract

The title compound, C_12_H_9_IN_2_OS, has an overall U-shape, with a dihedral angle of 21.4 (3)° between the thio­phene and benzene rings. In the crystal, supra­molecular chains mediated by N—H⋯O hydrogen bonds are formed along the *b*-axis direction.

## Related literature

For background to 2-substituted thio­phenes, see: Campaigne (1984 ▶); Kleemann *et al.* (2006 ▶). For the anti­mycobacterial activity of 2-substituted thio­phenes, see: Lourenço *et al.* (2007 ▶). For a related structure, see: Ferreira *et al.* (2009 ▶). For background to the production of mono-hydrazones by the reaction of aryl­hydrazines with arenedicarbaldehydes, see: Reuch & Heflet (1956 ▶); Vaysse & Pastour (1964 ▶); Butler *et al.* (1990 ▶); Glidewell *et al.* (2005 ▶); Low *et al.* (2006 ▶); Wardell *et al.* (2006 ▶).
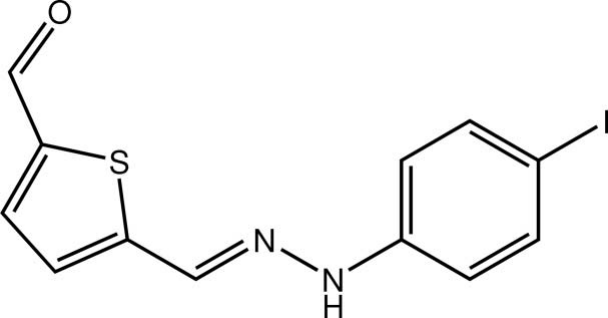


## Experimental

### 

#### Crystal data

C_12_H_9_IN_2_OS
*M* *_r_* = 356.17
Orthorhombic, 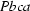

*a* = 6.9291 (9) Å
*b* = 11.7602 (10) Å
*c* = 30.958 (4) Å
*V* = 2522.7 (5) Å^3^
*Z* = 8
Mo *K*α radiation
μ = 2.69 mm^−1^
*T* = 120 K
0.16 × 0.08 × 0.05 mm

#### Data collection

Nonius KappaCCD diffractometer
Absorption correction: multi-scan (*SADABS*; Sheldrick, 2007 ▶) *T*_min_ = 0.616, *T*_max_ = 0.746
15735 measured reflections
2614 independent reflections
1674 reflections with *I* > 2σ(*I*)
*R*_int_ = 0.095

#### Refinement

*R*[*F* ^2^ > 2σ(*F* ^2^)] = 0.058
*wR*(*F* ^2^) = 0.113
*S* = 1.04
2614 reflections
157 parameters
1 restraint
H atoms treated by a mixture of independent and constrained refinement
Δρ_max_ = 1.08 e Å^−3^
Δρ_min_ = −0.60 e Å^−3^

### 

Data collection: *COLLECT* (Hooft, 1998 ▶); cell refinement: *DENZO* (Otwinowski & Minor, 1997 ▶) and *COLLECT*; data reduction: *DENZO* and *COLLECT*; program(s) used to solve structure: *SHELXS97* (Sheldrick, 2008 ▶); program(s) used to refine structure: *SHELXL97* (Sheldrick, 2008 ▶); molecular graphics: *ORTEP-3* (Farrugia, 1997 ▶) and *DIAMOND* (Brandenburg, 2006 ▶); software used to prepare material for publication: *publCIF* (Westrip, 2010 ▶).

## Supplementary Material

Crystal structure: contains datablocks global, I. DOI: 10.1107/S1600536809055172/hb5284sup1.cif

Structure factors: contains datablocks I. DOI: 10.1107/S1600536809055172/hb5284Isup2.hkl

Additional supplementary materials:  crystallographic information; 3D view; checkCIF report

## Figures and Tables

**Table 1:** Hydrogen-bond geometry (Å, °)

*D*—H⋯*A*	*D*—H	H⋯*A*	*D*⋯*A*	*D*—H⋯*A*
N1—H1n⋯O1^i^	0.88 (4)	2.05 (5)	2.916 (8)	172 (5)

Symmetry code: (i) 
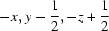
.

**Fig. 1.**
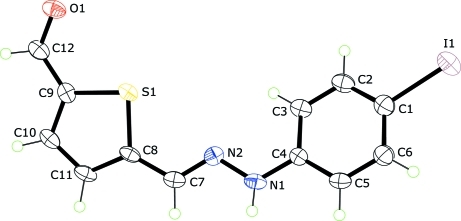
The molecular structure of (I) showing displacement ellipsoids at the 50% probability level.

**Fig. 2.**
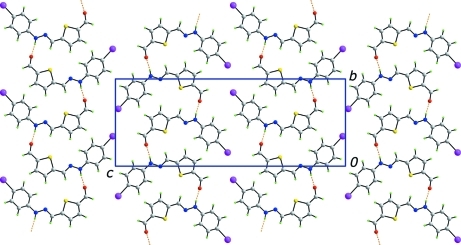
A view of the supramolecular 2-D array in (I) mediated by N–H···O hydrogen bonding (orange dashed lines) leading to chains in the b direction held together in the c direction by I···I contacts (not illustrated). Colour code: I, pink; S, yellow; O, red; N, blue; C, grey; and H, green.

**Fig. 3.**
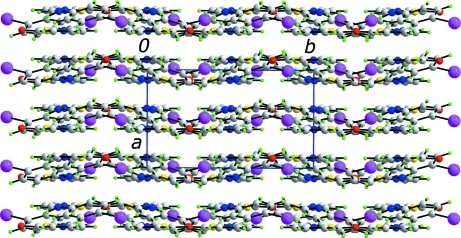
A view of the stacking of layers (illustrated in Fig. 2) along the a direction in (I). Colour code: I, pink; S, yellow; O, red; N, blue; C, grey; and H, green.

## supplementary crystallographic information

### Comment

The various uses of 2-substituted thiophenes have been well documented (Campaigne, 1984; Kleemann *et al.*, 2006). Amongst these appplications, are antimycobacterial activities, as found for a series of *N*-(aryl)-2-thiophen-2-ylacetamide derivatives (Lourenço *et al.*, 2007), one structure of which, *i.e. N*-(2,6-dimethylphenyl)-2-(thiophen-2-yl)acetamide, was recently reported (Ferreira *et al.*, 2009). Herein, we now report the structure of the title compound (I) prepared by the controlled reaction of *p*-iodophenylhydrazine with 2,5-thiophenedicarbaldehyde. As indicated in the literature, controlled reactions of arylhydrazines with arenedicarbaldehydes can successfully produce mono-hydrazones (Reuch & Heflet, 1956; Vaysse & Pastour, 1964; Butler *et al.*, 1990; Glidewell, *et al.*, 2005; Low *et al.*, 2006; Wardell *et al.*, 2006).

The overall molecule of (I), Fig. 1, is non-planar as evidenced by the dihedral angle of 21.4 (3)° formed between the thiophene and benzene rings. The twist in the molecule is most evident in the C4–N1–N2–C7 torsion angle of -172.2 (6) ° and. more particularly, in the adjacent N2–N1–C4–C3 torsion angle of -20.8 (9) °. The conformation about the C7═N2 bond [1.284 (8) Å] is *E*. The thiophene-S and aldehyde-O atoms are *syn* and are directed towards the benzene ring so that, overall, the molecule has a U-shape. The most prominent intermolecular interactions operating in the crystal structure are N–H···O hydrogen bonds, Table 1. These lead to supramolecular chains along the *b* direction, Fig. 2. Chains are connected along the *c* direction by I···I contacts [I···I^i^ = 3.7630 (8) Å for i: -*x*, 1 - *y*, 1 - *z*] to form a 2-D array. Layers thus formed stack along the *a* direction, Fig. 3.

### Experimental

A solution of *p*-iodophenylhydrazine (117 mg, 0.5 mmol) in MeOH (10 ml) was slowly added to a solution of 2,5-thiophenedicarbaldehyde (70 mg, 0.5 mmol) in MeOH (5 ml) at room temperature. The reaction mixture was maintained at room temperature and the crystals, which slowly formed, were collected and recrystallized from MeOH, m.pt. 464–467 K (dec.). IR(KBr, cm^-1^): 1675 (C═O), 1593(C═N).

### Refinement

The C-bound H atoms were geometrically placed (C–H = 0.95 Å) and refined as riding with *U*_iso_(H) = 1.2*U*_eq_(C). The N–H atom was located in a difference map and refined with the distance restraint N–H = 0.88±0.01 and with *U*_iso_(H) = 1.2*U*_eq_(N).

### Crystal data

**Table:**

C_12_H_9_IN_2_OS	*F*(000) = 1376
*M**_r_* = 356.17	*D*_x_ = 1.876 Mg m^−^^3^
Orthorhombic, *P**b**c**a*	Mo *K*α radiation, λ = 0.71073 Å
Hall symbol: -P 2ac 2ab	Cell parameters from 16555 reflections
*a* = 6.9291 (9) Å	θ = 2.9–27.5°
*b* = 11.7602 (10) Å	µ = 2.69 mm^−^^1^
*c* = 30.958 (4) Å	*T* = 120 K
*V* = 2522.7 (5) Å^3^	Prism, colourless
*Z* = 8	0.16 × 0.08 × 0.05 mm

### Data collection

**Table:**

Nonius KappaCCD diffractometer	2614 independent reflections
Radiation source: Enraf Nonius FR591 rotating anode	1674 reflections with *I* > 2σ(*I*)
10 cm confocal mirrors	*R*_int_ = 0.095
Detector resolution: 9.091 pixels mm^-1^	θ_max_ = 26.5°, θ_min_ = 3.2°
φ and ω scans	*h* = −8→8
Absorption correction: multi-scan (*SADABS*; Sheldrick, 2007)	*k* = −14→14
*T*_min_ = 0.616, *T*_max_ = 0.746	*l* = −38→37
15735 measured reflections	

### Refinement

**Table:**

Refinement on *F*^2^	Primary atom site location: structure-invariant direct methods
Least-squares matrix: full	Secondary atom site location: difference Fourier map
*R*[*F*^2^ > 2σ(*F*^2^)] = 0.058	Hydrogen site location: inferred from neighbouring sites
*wR*(*F*^2^) = 0.113	H atoms treated by a mixture of independent and constrained refinement
*S* = 1.04	*w* = 1/[σ^2^(*F*_o_^2^) + (0.0257*P*)^2^ + 17.1487*P*] where *P* = (*F*_o_^2^ + 2*F*_c_^2^)/3
2614 reflections	(Δ/σ)_max_ = 0.001
157 parameters	Δρ_max_ = 1.08 e Å^−^^3^
1 restraint	Δρ_min_ = −0.60 e Å^−^^3^

### Special details

**Table:**

Geometry. All s.u.'s (except the s.u. in the dihedral angle between two l.s. planes) are estimated using the full covariance matrix. The cell s.u.'s are taken into account individually in the estimation of s.u.'s in distances, angles and torsion angles; correlations between s.u.'s in cell parameters are only used when they are defined by crystal symmetry. An approximate (isotropic) treatment of cell s.u.'s is used for estimating s.u.'s involving l.s. planes.
Refinement. Refinement of *F*^2^ against ALL reflections. The weighted *R*-factor *wR* and goodness of fit *S* are based on *F*^2^, conventional *R*-factors *R* are based on *F*, with *F* set to zero for negative *F*^2^. The threshold expression of *F*^2^ > 2σ(*F*^2^) is used only for calculating *R*-factors(gt) *etc*. and is not relevant to the choice of reflections for refinement. *R*-factors based on *F*^2^ are statistically about twice as large as those based on *F*, and *R*- factors based on ALL data will be even larger.

### Fractional atomic coordinates and isotropic or equivalent isotropic displacement parameters (Å^2^)

**Table:**

	*x*	*y*	*z*	*U*_iso_*/*U*_eq_	
I1	−0.01127 (8)	0.34335 (4)	0.487908 (18)	0.04277 (19)	
S1	0.1013 (3)	0.10271 (14)	0.21597 (6)	0.0261 (4)	
N1	0.0936 (8)	−0.0324 (5)	0.3466 (2)	0.0282 (14)	
H1N	0.039 (9)	−0.099 (3)	0.350 (2)	0.034*	
N2	0.1043 (8)	0.0033 (5)	0.3048 (2)	0.0266 (13)	
O1	0.1012 (7)	0.2541 (4)	0.13359 (16)	0.0336 (12)	
C1	0.0410 (10)	0.2143 (6)	0.4423 (2)	0.0323 (18)	
C2	0.1194 (10)	0.2425 (6)	0.4030 (2)	0.0322 (18)	
H2	0.1610	0.3182	0.3976	0.039*	
C3	0.1374 (9)	0.1609 (6)	0.3714 (2)	0.0278 (16)	
H3	0.1900	0.1808	0.3441	0.033*	
C4	0.0794 (9)	0.0499 (6)	0.3792 (2)	0.0231 (15)	
C5	0.0110 (10)	0.0198 (5)	0.4197 (2)	0.0284 (16)	
H5	−0.0202	−0.0573	0.4257	0.034*	
C6	−0.0121 (10)	0.1017 (6)	0.4514 (2)	0.0332 (17)	
H6	−0.0631	0.0820	0.4789	0.040*	
C7	0.0949 (10)	−0.0729 (6)	0.2752 (2)	0.0275 (17)	
H7	0.0837	−0.1509	0.2827	0.033*	
C8	0.1016 (9)	−0.0394 (5)	0.2306 (2)	0.0249 (16)	
C9	0.1085 (9)	0.0672 (6)	0.1616 (2)	0.0239 (15)	
C10	0.1118 (10)	−0.0491 (6)	0.1562 (2)	0.0291 (17)	
H10	0.1177	−0.0849	0.1288	0.035*	
C11	0.1057 (10)	−0.1090 (6)	0.1951 (2)	0.0292 (16)	
H11	0.1046	−0.1897	0.1968	0.035*	
C12	0.1088 (9)	0.1509 (6)	0.1282 (2)	0.0288 (16)	
H12	0.1156	0.1243	0.0993	0.035*	

### Atomic displacement parameters (Å^2^)

**Table:**

	*U*^11^	*U*^22^	*U*^33^	*U*^12^	*U*^13^	*U*^23^
I1	0.0425 (3)	0.0365 (3)	0.0493 (3)	−0.0005 (3)	0.0024 (3)	−0.0128 (3)
S1	0.0228 (9)	0.0219 (8)	0.0336 (10)	−0.0006 (8)	0.0004 (8)	−0.0049 (8)
N1	0.023 (3)	0.020 (3)	0.042 (4)	−0.002 (3)	0.004 (3)	0.001 (3)
N2	0.020 (3)	0.030 (3)	0.030 (4)	0.001 (3)	0.005 (3)	0.003 (3)
O1	0.027 (3)	0.025 (3)	0.049 (3)	−0.003 (2)	−0.004 (3)	−0.001 (2)
C1	0.029 (4)	0.033 (4)	0.035 (4)	0.001 (3)	−0.007 (3)	−0.003 (3)
C2	0.025 (4)	0.027 (4)	0.045 (5)	−0.005 (3)	−0.005 (4)	−0.002 (3)
C3	0.018 (3)	0.026 (4)	0.039 (4)	−0.002 (3)	−0.002 (3)	−0.001 (3)
C4	0.018 (3)	0.024 (4)	0.027 (4)	0.001 (3)	−0.002 (3)	−0.001 (3)
C5	0.027 (4)	0.020 (3)	0.038 (4)	0.001 (3)	−0.003 (4)	0.003 (3)
C6	0.034 (4)	0.032 (4)	0.034 (4)	0.002 (4)	0.001 (4)	−0.001 (3)
C7	0.021 (4)	0.022 (4)	0.040 (5)	0.000 (3)	0.000 (4)	0.001 (3)
C8	0.017 (3)	0.018 (3)	0.039 (4)	−0.002 (3)	0.001 (3)	−0.005 (3)
C9	0.012 (3)	0.031 (4)	0.029 (4)	0.002 (3)	0.001 (3)	−0.006 (3)
C10	0.023 (4)	0.025 (4)	0.039 (5)	0.001 (3)	−0.002 (3)	−0.011 (3)
C11	0.026 (4)	0.018 (3)	0.044 (5)	0.000 (3)	0.010 (4)	−0.006 (3)
C12	0.020 (3)	0.027 (4)	0.040 (5)	0.005 (4)	−0.002 (3)	−0.007 (3)

### Geometric parameters (Å, °)

**Table:**

I1—C1	2.103 (7)	C4—C5	1.387 (9)
S1—C8	1.732 (7)	C5—C6	1.386 (9)
S1—C9	1.734 (7)	C5—H5	0.9500
N1—N2	1.362 (8)	C6—H6	0.9500
N1—C4	1.402 (8)	C7—C8	1.436 (9)
N1—H1N	0.876 (10)	C7—H7	0.9500
N2—C7	1.284 (8)	C8—C11	1.371 (9)
O1—C12	1.227 (8)	C9—C10	1.378 (9)
C1—C2	1.374 (10)	C9—C12	1.429 (9)
C1—C6	1.403 (10)	C10—C11	1.395 (10)
C2—C3	1.376 (9)	C10—H10	0.9500
C2—H2	0.9500	C11—H11	0.9500
C3—C4	1.388 (9)	C12—H12	0.9500
C3—H3	0.9500		
			
C8—S1—C9	91.2 (3)	C5—C6—H6	120.5
N2—N1—C4	118.3 (6)	C1—C6—H6	120.5
N2—N1—H1N	114 (5)	N2—C7—C8	119.6 (6)
C4—N1—H1N	120 (5)	N2—C7—H7	120.2
C7—N2—N1	117.4 (6)	C8—C7—H7	120.2
C2—C1—C6	120.6 (7)	C11—C8—C7	127.4 (6)
C2—C1—I1	119.2 (5)	C11—C8—S1	111.5 (5)
C6—C1—I1	120.1 (5)	C7—C8—S1	121.1 (5)
C1—C2—C3	119.8 (7)	C10—C9—C12	126.6 (6)
C1—C2—H2	120.1	C10—C9—S1	110.9 (5)
C3—C2—H2	120.1	C12—C9—S1	122.5 (5)
C2—C3—C4	120.5 (7)	C9—C10—C11	113.3 (6)
C2—C3—H3	119.8	C9—C10—H10	123.3
C4—C3—H3	119.8	C11—C10—H10	123.3
C3—C4—C5	119.7 (6)	C8—C11—C10	113.0 (6)
C3—C4—N1	120.4 (6)	C8—C11—H11	123.5
C5—C4—N1	119.9 (6)	C10—C11—H11	123.5
C6—C5—C4	120.2 (6)	O1—C12—C9	125.7 (7)
C6—C5—H5	119.9	O1—C12—H12	117.2
C4—C5—H5	119.9	C9—C12—H12	117.2
C5—C6—C1	118.9 (7)		
			
C4—N1—N2—C7	−172.2 (6)	N2—C7—C8—C11	175.5 (7)
C6—C1—C2—C3	3.2 (11)	N2—C7—C8—S1	−5.6 (9)
I1—C1—C2—C3	−173.9 (5)	C9—S1—C8—C11	−0.4 (5)
C1—C2—C3—C4	−0.9 (10)	C9—S1—C8—C7	−179.4 (6)
C2—C3—C4—C5	−3.0 (10)	C8—S1—C9—C10	−0.3 (5)
C2—C3—C4—N1	178.4 (6)	C8—S1—C9—C12	179.2 (6)
N2—N1—C4—C3	−20.8 (9)	C12—C9—C10—C11	−178.5 (6)
N2—N1—C4—C5	160.6 (6)	S1—C9—C10—C11	0.9 (8)
C3—C4—C5—C6	4.5 (10)	C7—C8—C11—C10	180.0 (7)
N1—C4—C5—C6	−176.9 (6)	S1—C8—C11—C10	1.0 (8)
C4—C5—C6—C1	−2.1 (11)	C9—C10—C11—C8	−1.2 (9)
C2—C1—C6—C5	−1.7 (11)	C10—C9—C12—O1	178.2 (7)
I1—C1—C6—C5	175.4 (5)	S1—C9—C12—O1	−1.2 (10)
N1—N2—C7—C8	178.7 (6)		

### Hydrogen-bond geometry (Å, °)

**Table:**

*D*—H···*A*	*D*—H	H···*A*	*D*···*A*	*D*—H···*A*
N1—H1n···O1^i^	0.88 (4)	2.05 (5)	2.916 (8)	172 (5)

Symmetry codes: (i) −*x*, *y*−1/2, −*z*+1/2.
